# Supplementary material for: Are carbon emissions trading and green financial instruments synergistic? -Comprehensive quantitative research based on content analysis
Source: PLoS One. 2024 Mar 7;19(3):e0298601. doi: 10.1371/journal.pone.0298601 (PMC10919601; doi:10.1371/journal.pone.0298601)
Supplement: S1 Data — (ZIP) [file pone.0298601.s001.zip › Data/地级市Z_F工作报告.zip!/╜¡╬≈/─╧▓2╩╨/└┤╫╘╣1⁄2╓┌║┼í╛┬φ┐╦╩2╛▌═°í┐_2018.docx]

　一、2017年工作回顾

　　各位代表：

　　现在，我代表市人民政府向大会报告工作，请予审议，并请各位市政协委员和列席会议的同志提出意见。

　　一、2017年工作回顾

　　过去一年，在省委、省政府和市委的坚强领导下，全市上下深入学习贯彻习近平新时代中国特色社会主义思想，主动适应把握经济发展新常态，坚持稳中求进工作总基调，排难而进，奋发图强，较好完成了市十五届人大二次会议确定的目标任务，打造富裕美丽幸福现代化江西“南昌样板”取得明显成效。

　　——综合实力迈上新台阶。全年完成地区生产总值5003.2亿元，增长9%左右；财政总收入（市口径）845.3亿元，增长6.9%；地方一般公共预算收入417.1亿元，同口径增长10.3%；规模以上工业增加值增长9.5%左右；全社会固定资产投资5115.2亿元，增长12.7%；实际利用内资1390亿元，增长20%；实际利用外资31.8亿美元，增长10.1%；社会消费品零售总额2097亿元，增长12.4%左右；外贸出口63亿美元，增长8.9%。其中地区生产总值、规模以上工业增加值、固定资产投资、实际利用外资、社会消费品零售总额等五项指标增速稳居全国省会城市第一方阵，地区生产总值和规模以上工业增加值两项指标增速位居中部省会城市首位，财政六项指标位居全省第一，规模以上工业增加值首次位居全省第一，外贸出口增速实现由负转正，打了翻身仗。

　　——区域发展竞相发力。高新、经开、小蓝三个国家级开发区主营业务收入分别实现2100亿元、1250亿元、1200亿元。西湖区、红谷滩新区财政总收入首次突破100亿元大关。高新区综合排名位居157个国家级高新区第40位；经开区综合排名位居219个国家级经开区第47位。南昌县在全国中小城市综合实力百强县、投资潜力百强县、新型城镇化质量百强县排位前移；青山湖区、西湖区入选全国投资潜力百强区。

　　——“天下英雄城”品牌不断唱响。纪念八一南昌起义暨建军90周年系列活动成功举办，八一广场、军事雕塑广场等一批标志性建筑全新亮相，第五届国际军乐节规模、档次实现新突破，电影《建军大业》在全国火爆上映并斩获第十四届“五个一工程”特别奖，“天下英雄城”形象片以强烈的视觉冲击惊艳海内外；开通旅游专列132趟次、境外包机75架次，“天下英雄城”品牌知名度和影响力空前提升。

　　——民生福祉持续改善。2016年全面建成小康监测指数达到94.56%，位居全省第一。全年居民消费价格总水平涨幅2.1%，低于预期目标。全市城镇化率达73%，高于全国和全省平均水平。城乡居民人均可支配收入分别实现37675元、16364元，增长8.8%、9.4%。

　　过去一年，我们主要抓好了以下几方面重点工作：

　　（一）强攻产业、决战工业，实体经济加速壮大

　　现代制造业量质双升。106个重大重点工业项目顺利推进，预计全市完成工业投资1800亿元，增长11%左右。电子信息产业爆发式增长。预计主营业务收入突破800亿元，增长30%以上。“南昌光谷”建设加速，移动智能终端产业呈井喷态势，华勤、闻泰、与德、龙旗、天珑等全国手机ODM企业前五强均已落户，全市已拥有整机生产企业9家、手机产业链生产企业30余家，零部件配套率达90%以上；LED产业加速壮大，新增深圳兆驰LED外延片和芯片、乾照光电南昌基地、艾立特光电南昌LED封装生产线、华夏光彩户外LED显示屏南昌生产基地等一大批项目，以硅衬底LED核心技术和MOCVD关键核心装备为支撑的LED产业实现裂变式的扩张。汽车和新能源汽车产业加快发展。预计主营业务收入突破千亿，增长20%。经开区江铃集团30万辆新能源汽车基地、江铃股份富山新能源汽车基地开工建设。江西凯马百路佳混合动力客车和纯电动客车列入国家新能源汽车目录。生物医药产业稳中有进。预计主营业务收入增长13.5%左右。全市药品生产占全省总量的30%，中成药生产规模居全国城市前列；医疗器械生产占全省总量的60%，一次性输注器械制造规模全国最大。中国（南昌）中医药科创城加快建设，济民可信生物医药产业园顺利落户。航空产业平台做实。成功获批全国首批通用航空产业综合示范区。南昌航空城建设加速推进，瑶湖机场竣工验收、即将启用。传统产业加快转型。全市实施500万元以上技术更新和改造项目174个。产业集群不断壮大。小蓝汽车及零部件产业集群主营业务收入突破450亿元，高新区光电及通信产业集群达400亿元左右，经开区光电产业集群、青山湖区针织服装产业集群均突破300亿元。龙头企业加速壮大。预计江铃集团主营业务收入突破800亿元，欧菲光达到280亿元，双胞胎集团、正邦科技突破200亿元，方大特钢、智慧海派突破100亿元。预计全市规模以上企业新增200家以上，总数超过1400家。

　　现代服务业繁荣活跃。44个重大重点服务业项目顺利推进，预计完成第三产业投资3235亿元，增长13.8%。VR产业加速发力。中国（南昌）VR 产业基地一期建成投用，引进了联想新视界、北京理工大学等一大批龙头企业和研究机构；VR产业国家标准检测、云服务、技术研究、天使基金、产品交易、基地线上等“六大平台”初步建成；VR体验中心和产业展示中心等标志性示范项目加速建设；VR产业省级创新中心通过正式验收。金融业态势喜人。全市金融机构本外币存贷款余额双双突破万亿大关。全省金融商务区快速发展，全国首家省级互联网金融产业园、江西基金产业园先后成立，汇集各类金融机构超700家。多层次资本市场稳步发展，新增联创电子、普天通信2家上市公司，境内外上市公司达25家，新三板挂牌公司突破50家，江西联合股权交易中心挂牌企业突破1000家，直接融资金额突破千亿大关。积极防范化解地方金融风险，保持打击非法集资高压态势，全市金融市场秩序保持稳定。商贸物流业快速发展。杉杉奥特莱斯购物广场、沃尔玛南昌乐世界购物中心、铜锣湾广场等商贸综合体顺利开业。电子商务快速发展，预计全年相关交易额突破2000亿元，增长30%。预计新增服务外包企业100家，服务贸易额33亿美元，增长16%以上，连续三次被评为“中国服务外包中西部最具竞争力城市”。全国物流标准化试点、国家智慧物流配送示范城市建设进展顺利，向塘综合物流产业集群被认定为全省唯一省级物流中心，南昌区域物流中心城市地位显著增强。旅游业品质提升。预计完成旅游接待总人数1.2亿人次、旅游综合收入1200亿元，分别增长44.4%、46.9%。南昌汉代海昏侯国遗址公园列入第三批国家考古遗址公园立项名单。滕王阁景区创5A即将通过国家验收审批；万达文化旅游城、安义古村群创4A通过国家评审。湾里区获评全国森林旅游示范县（区）。成功举办各类展会336场次，会展营收实现翻番。

　　都市现代农业提质增效。完成农林牧渔及服务业总产值312亿元，同比增长3.6%左右。南昌绿谷“两区一廊”建设扎实推进，溪霞现代农业园示范区二期全面启动建设，江西省现代农业博览园、赣鄱珍奇植物园、铭宸智慧农业园等项目相继落户。中国汉光集团农产品加工冷链智能物流园、市政控股集团南新农业生态园、昌工控股集团塘南菜篮子农业园等一批现代农业项目开工建设。大力实施“一乡一园”战略，现代农业园区总数达106个，总投资达37亿元，建设面积10.65万亩，总产值达39亿元，园区规模效益全面提升。农业产业化加快推进，全市国家级农业产业化龙头企业12家，占全省的三分之一，预计市级以上龙头企业销售收入达到1718亿元。大力推动现代农业多元化发展，积极培育和发展农村电商、休闲农业、民宿经济等新型业态，全市发展涉农电子商务销售企业3120家，销售额突破20亿元。

　　（二）统筹推进、建管并重，城乡面貌大为改观

　　“大南昌”框架拉开。城市规划加快完善。《南昌大都市区规划》已经完成，新一轮《城市总体规划》修编形成纲要方案，《南昌市空间规划》（多规合一）初步方案规划工作稳步推进。《海绵城市专项规划》《梅岭及周边地区总体规划》等专项规划加快编制。立体交通加快建设。红谷隧道、沿江大道南延工程、南外环迎宾互通以西段、昌九快速路改造一期、沿江北大道快速路均已通车；前湖大道快速路、志敏大道改造、昌西大道、洪都大道快速路改造、昌南大道快速路改造等项目扎实推进，以“十横十纵”干线路网为基础的“三环”路网体系基本成型。地铁2号线首通段已开通运营，地铁3号线、4号线扎实推进。昌景黄铁路南昌东站、进贤军山湖站工程可行性研究报告已上报国家发改委，南昌至抚州、南昌至修水城际铁路已开展前期研究。《南昌港总体规划》加快修编，龙头岗综合码头一期建成投入使用。昌北国际机场T1航站楼正在实施升级改造，T2航站楼扩建加快启动，年旅客吞吐量突破1000万人次，成为全国第31个千万级枢纽机场。配套功能均衡布点。九龙湖花园、朝阳新城等8个新农贸市场竣工。新增社区达标便利店200家。建成公共停车场41个，新增泊位数12516个。实验中学高新校区、汽车机电学校、中小学生校外实践基地、市第三医院朝阳院区、市第一医院九龙湖分院等项目加快推进。建设品质不断提升。推进海绵城市项目18个，建成面积15.98平方公里。九龙湖城市地下综合管廊一期工程已开工建设，洛阳东路综合管廊项目、青山北路综合管廊项目扎实推进。装配式建筑发展快速起步，2家产业基地被评为全国第一批装配式建筑产业基地。江西检验检疫局综合实验用房项目、中大城（7号、8号楼）项目被评为“鲁班奖”，地铁1号线、南昌万达茂项目被评为国家优质工程奖。

　　城乡环境显著提升。“美丽南昌·幸福家园”城乡环境综合整治三年行动精彩开局，“一年有变化”的目标基本实现。城市更整洁了。以“六改”为重点的城市容貌提升工程顺利推进，完成111条道路“白改黑”、122个点位“暗改亮”、3条道路强弱电管线“上改下”、5个城市出入口“堵改通” 、32个老旧社区进行“旧改新”；“面改美”成效明显，城市主干道门店招牌达标率95%以上。“城市家具微更新”试点进展顺利。城市更干净了。“马路本色”行动顺利实施，保洁经费标准得到提高，保洁基础设施更加完善，机械化清扫、人行道冲洗、快速流动保洁的“立体全覆盖保洁法”加快推广，全市主次干道路面净化得到稳定和巩固，背街小巷、社区环境卫生大幅改善。城市更畅通了。“畅通省城”行动继续深入，市政道路改造及桥梁加固各项工程快速推进，城市道路、桥梁安全隐患有效排除。公交都市创建工程加快推进，首条老福山至银三角BRT1号线投入运行。门店装修、门店经营、道路开挖、施工围挡、施工现场冲洗、施工车辆治超、非机动车停放等整治有力、规范有序。“六项”违法违章交通行为的自动抓拍机制，被公安部列为先进典型在全国推广。城市更文明了。文明出行、志愿服务、养犬管理、烟花爆竹禁燃、文明祭扫等活动初见成效。全市“最美人物”中的13人荣登“中国好人榜”。顺利蝉联“国家卫生城市”。南昌县荣获“国家卫生县城”称号和全省首个“全国县级文明城市”称号。乡村更美丽了。按照“拆、清、绿、改、装、亮”的要求，开展了7条铁路沿线环境整治。5个农业县区率先全面通过全省农村生活垃圾治理第三方评估考核验收，全市农村生活垃圾处理率达95%。8个百强中心镇、45个村点水环境治理扎实开展。

　　村镇建设稳步推进。扎实推进新农村建设三年“扫一遍”，各县区高标准编制了村镇建设的全域规划，完成了2291个新农村村点整治。全面开展“六统·四联创”示范创建，推进了安义县全省美丽示范县、南安公路等10条美丽示范带、蒋巷镇等6个示范乡镇以及32个示范村点和近5000个示范庭院创建工作。推进了年度21个特色小镇、30个“五位一体”综合示范村、15个农村新户型推广示范村建设。太平镇、文港镇入选为全国特色小镇。溪霞镇、李渡镇、太平镇、文港镇获评江西省第一批特色小镇。黄马乡成功申报全国“一村一品”示范村镇。湾里畅安舒美示范路工程全线完工，万埠至八景公路灾毁恢复重建工程加快推进。进贤县、新建区荣获“四好农村路”省级示范县。

　　（三）倡导绿色、守住底线，生态治理攻坚破局

　　绿色发展水平持续攀升。全年空气质量优良率83%，PM10实现“双低”和“双最”目标，连续四年在中部省会城市排名第一。赣江、抚河南昌段14个地表水断面水质达标率100%。城区集中式饮用水水源地水质达标率99.5％。土壤环境质量总体保持稳定。预计万元GDP能耗下降3.5%，公共机构节能走在全省前列。完成绿色建筑设计标识项目71个，建筑面积867万平方米，绿色建筑占新建建筑50%，占全省绿色建筑81%。

　　生态环境工程稳步推进。中央环保督察反馈问题整改扎实推进，“蓝天、清流、净土”行动深入实施。大气污染防治机制更加完善，生态环境大数据平台及多模式空气质量预报预警项目进展顺利，工业废气、城市扬尘、锅炉烟气、机动车排气污染整治有力，秸秆禁烧及综合利用全面推行。全面完成幸福二支河、西湖孺子亭公园等黑臭水体治理。湾里区全域划定为畜禽养殖禁养区，提前7个月率先退出生猪养殖。青山湖电排站等劣Ⅴ类水断面水质治理、赣抚尾闾综合整治、环鄱阳湖综合整治等工程稳步推进。工业企业水污染防治管理加强。市本级开展环境保护执法行动出动执法人员9930人次，平均每天近30人次。

　　生态文明体系不断健全。出台了全面落实主体责任、领导干部自然资源资产离任审计、控制污染物排放许可制实施计划、畜禽养殖粪污集中处理及资源化利用等制度性文件。编制了《城市水环境治理综合规划》，“河湖长制”全面升级。林业生态体系和林业保护体系进一步完善。国家水生态文明城市建设试点通过水利部评估验收，成功获评“全国水生态文明城市”。启动了梅岭国家森林公园全省首批重要生态区非国有商品林赎买试点工作。南昌县、安义县、湾里区、新建区省级生态文明先行示范县（区）建设全面推进。

　　（四）改革创新、开放带动，内生动力全面激活

　　创新驱动战略深入实施。国家创新型城市建设深入推进，预计全市R&D经费占GDP比重1.65%；专利授权量9000件，增长4.7%；新认定国家高新技术企业超过300家，总数突破600家；完成技术合同登记额42.8亿元，增长9.38%；成功获批建设中国（南昌）知识产权保护中心；新增国家企业技术中心1家；新增省级重点实验室1家、省级工程技术研究中心4家、市级重点实验室7家、市级工程技术研究中心14家；天津大学微技术研究院、哈尔滨工业大学航天科创中心、北京理工大学VR创新中心、中科院纳米研究院等协同创新平台加快建设。国家“两创”示范城市建设成果丰硕，成功举办第六届中国创新创业大赛江西赛区暨第二届“洪城之星”创新创业大赛，建成各类众创空间92家，其中国家备案23家，省级众创空间32家；省级创业孵化基地数量跃居全省前列。全面实施各类人才计划，其中“洪城海鸥计划”引进外国专家项目20个、留学人才团队项目10个、个人项目10个。中国南昌人力资源服务产业园被列为全国第10个国家级人力资源服务产业园。全省首家、全国第7家中国青年创业社区落户小蓝经开区。

　　全面深化改革成效显著。构建开放型经济新体制综合试点试验成效突出，“重资本轻资产”招商模式、大数据市场主体监管新模式和推行国税地税联合办税三项创新举措，作为首批试点经验被商务部等十三部委向全国推广。供给侧结构性改革纵深推进，“三去一降一补”任务全面落实，全面降低企业用地、用工、用能、融资、物流和税收成本，为企业减负300多亿元。“放管服”改革深入推进，分两批取消和调整市级行政权力事项206项，规范行政审批中介服务，推进行政许可标准化建设，推动政务服务事项集中办理。开展相对集中行政许可权改革试点，组建了南昌市行政审批局，真正实现了“一枚印章管审批”。投融资体制改革取得突破，组建了规模达1231亿元的市级产业发展基金。供销综合改革走在全省前列。农村土地确权登记颁证工作全面完成。农村集体产权制度改革试点、建设用地二级市场试点、自然资源统一确权登记试点、科技和金融结合试点等工作进展顺利；事业单位改革、国资国企改革、农垦改革、出租车行业改革、司法体制改革及公证机构体制改革工作稳步推进。

　　双向开放步伐明显加快。积极融入“一带一路”建设，参与印尼、马来西亚等20多个“一带一路”沿线国家的国际产能合作。深度融入长江经济带发展，联合签署实施了《长江中游城市群省会城市合作行动计划（2017-2020年）》，共建全国首个跨区域劳动监察网上协查平台。“侨梦苑”平台更加夯实。南昌综合保税区封关运行，南昌（向塘）至福州（江阴港）、南昌（向塘）至宁波（舟山港）、南昌（向塘）至深圳（盐田港）海铁联运外贸货运班列和首列赣欧（亚）南昌至河内国际货运班列相继开行，外贸进出口集装箱运量呈现爆发式增长，预计增长600%。组织和参与各类招商推介活动101场，共引进重大项目620个，计划总投资4000亿元。

　　（五）共建共享、补齐短板，群众获得感显著提升

　　脱贫攻坚战全面打响。贫困村新农村建设、农村环境综合整治、光伏扶贫工程“三个全覆盖”和“低保兜底零遗漏、医疗服务零障碍、教育保障零负担、危房改造零压力”全面实现，“政府帮、部门助、企业扶”多元结对帮扶机制全面建立。年度脱贫攻坚任务圆满完成，预计贫困发生率从2%下降到0.66%。

　　民生保障全力以赴。投入487.9亿元用于民生及社会公益事业支出，占公共财政总支出比重达74.6%，比2016年提高2.1个百分点。全市城镇就业率达94.9%，零就业家庭就业安置率达100%。城镇养老、医疗、失业、工伤、生育五大保险参保人数再创新高。在全省率先实现城区低保城乡统筹，城区、县城、农村低保标准分别由510元、480元、310元提高到560元、530元、345元。城乡医疗救助比例显著提升，三无常补对象、特困供养人员救助达100%。强化房地产调控和市场管理，出台了“限贷、限购、限售、限价”政策，实施了“限房价、竞地价”政策，房地产市场保持平稳健康运行。提前2个月完成了省里下达的25142户城市棚户区改造任务，老旧城区连线成片城镇棚户区改造基本完成，公租房分配目标任务超额完成，中心城区31个集中安置小区加快建设。

　　社会事业全面进步。新建各类幼儿园91所，推进县区义务教育网点建设43个，建设改造农村义务教育学校面积70.75万平方米。安义县、高新区顺利通过义务教育均衡发展国家督导评估。南昌师范高等专科学校正式升格为豫章师范学院，实现了市属本科院校零的突破。成人高等教育资源整合有序推进。城市公立医院综合改革全面推开，彻底终结了我市持续半个多世纪的“以药补医”现状。在全省率先实现跨省异地就医住院费用直接结算以及“特殊家庭”签约服务和人员保险全覆盖。居家和社区养老服务改革试点工作顺利通过国家绩效考核。南昌国际马拉松、“千里跑赣鄱”乡村马拉松、全国三人制篮球锦标赛等活动成功举办，“八一女篮”正式落户南昌。文化消费试点工作稳步推进。南昌市图书馆等4个图书馆被评为国家一级图书馆。街道文化活动中心实现全覆盖。“弃婴救治”等7个项目荣获江西省首届“赣鄱慈善奖”。国家食品安全示范城市创建工作顺利推进。此外，国防动员、人民防空、民族宗教、史志档案、粮食供销、气象地震、防灾减灾、国税地税、金融保险、机关事务、老龄慈善、保密、参事、残疾人、职工、青少年、妇女儿童等各项事业也取得新成绩。

　　（六）创新治理、从严治政，行政效能极大改善

　　社会治理不断创新。深化平安南昌建设，积极推进“雪亮工程”建设，新增高清探头11800余个，严厉打击暴力刑事犯罪和“盗抢骗”侵财犯罪，发案率显著降低，破案率持续提升，市民安全感显著增强。深化“1341”戒毒模式，在全省首创社区矫正“队建制”模式。深化少数民族流动人口服务管理，阿卜杜拉·吾拉西木一封饱含各民族“情同手足、守望相助”的家书，在全国引起强烈反响。妥善应对各类突发事件，应急处置能力迈上新台阶。积极开展信访积案化解攻坚，基层矛盾化解机制更加健全。12345政府服务热线平台体系更加完善，全年接听群众有效来电335442次，工单按期办结率为91.46%，群众满意率为98.66%。

　　法治建设持续深化。全面推进“七五”普法，落实“谁执法谁普法”责任制。行政执法公示制度、执法全过程记录制度、重大执法决定法制审核制度试点工作顺利推进。自觉接受人大及其常委会法律监督、政协民主监督，高度重视舆论和社会监督，认真办理人大建议286件、政协提案511件；提请审议地方性法规草案1件，修改政府规章16件，废止政府规章17件。

　　作风建设持续推进。认真落实全面从严治党要求，深入推进“两学一做”学习教育常态化制度化，严格执行中央八项规定，坚决纠正“四风”，加强行政监察和审计监督，严惩庸懒散虚，作风效能问责2057人，立案查处违纪违法案件1202件，全市依法行政、阳光理政、高效勤政、廉洁从政水平进一步提高。

　　各位代表！这些成绩的取得，是党中央、国务院，省委、省政府和市委坚强领导的结果，是市人大、市政协和社会各界监督支持的结果，是全市广大干部群众团结奋斗的结果。在此，我谨代表南昌市人民政府，向全市人民，向所有关心支持南昌发展的同志们、朋友们，表示衷心的感谢，并致以崇高的敬意！

　　在总结成绩的同时，我们也要清醒地认识到，发展不平衡、不充分是我市的主要矛盾，综合经济实力与标兵城市差距较大；受政府融资难度加大、民间投资乏力等多重影响，全社会固定资产投资增速没有实现预期目标；科技创新驱动作用还不够显著，产业结构有待优化，新的经济增长动能有待加速培育；城乡公共服务供给不够均衡，各项民生设施配套总体不足，交通拥堵、环境污染、教育、医疗、文化、养老等短板亟待补齐；安全生产和社会治理存在不少隐患；少数干部担当精神不足，不作为、慢作为、隐形变异的“四风”现象还时有发生。对此，我们一定高度重视，坚持问题导向，强化担当使命，认真加以解决。

　　二、2018年主要工作

　　2018年是全面深入贯彻落实党的十九大精神的开局之年，是改革开放40周年，也是决胜全面建成小康社会、实施“十三五”规划承上启下的关键一年。我们要与新时代脉搏同频共振。朝着“两个一百年”伟大目标奋进，到2020年与全国同步全面建成小康社会，到2035年全市基本实现社会主义现代化，到本世纪中叶建成富强民主文明和谐美丽的社会主义现代化城市。我们要用新思想指导登高望远。高举习近平新时代中国特色社会主义思想伟大旗帜，坚持稳中求进工作总基调，抢抓质量变革、效率变革、动力变革战略机遇，推动南昌经济不断向高质量、高效率发展，以当仁不让的姿态在全省发展中先行先试、领先领跑，以担当拼搏的精神在全国省会城市中追赶进位、争先争优。我们要以新作风扛起使命担当。深入开展“不忘初心、牢记使命”专题教育，深入查摆、及时纠正“四风”问题，坚定不移全面从严治党，持之以恒正风肃纪，开拓创新善担当，快速反应破难题，雷厉风行抓落实，以优异成绩向改革开放40周年献礼。

　　今年政府工作的总体要求是: 以习近平新时代中国特色社会主义思想为指导，全面贯彻落实党的十九大、中央经济工作会、省委十四届五次全会和市委十一届四次全会精神，紧紧围绕建设“六区”要求，坚持以高质量发展为目标引领，坚持稳中求进工作总基调，坚持既定战略目标、总体布局、工作举措不动摇，着力抓重点、补短板、强弱项，深入推进强产业、兴城市、促改革、优生态、惠民生、重党建等各项工作，保持定力，精准发力，持续加力，不断开创打造富裕美丽幸福现代化江西“南昌样板”新局面。

　　今年经济社会发展的主要预期目标是：地区生产总值增长9%左右；财政总收入（省口径）增长8%左右；地方一般公共预算收入增长6%左右；全社会固定资产投资增长12%左右；规模以上工业增加值增长9%左右；社会消费品零售总额增长12%；实际利用外资增长10%；实际利用内资增长10%；出口总额增长3%；城镇居民人均可支配收入增长8%左右；农村居民人均可支配收入增长9%左右；万元生产总值能耗下降3.5%；居民消费价格总水平涨幅控制在3%左右。

　　今年政府将重点抓好以下几方面工作：

　　（一）牢牢把握新时代的发展趋势，坚持质量第一、效益优先，加快构建现代化经济体系

　　1.聚焦实体经济这个重点，不断厚植现代化经济发展的产业优势。

　　加快发展先进制造业。坚定不移推进“强攻产业、决战工业”， 不断提升工业经济质量和效益。培育壮大新兴产业。推动同济大学汽车创新研究院落地，加快经开区、小蓝经开区、新建区三大新能源汽车基地建设。加快“南昌光谷”建设，扩大硅衬底LED全球合伙人计划，全面拉长、增厚、拓宽以MOCVD高端装备为核心的光电产业全产业链；以华勤、与德等龙头企业为引领，加速形成移动智能终端全产业链，力争电子信息产业主营业务收入突破千亿。稳步推进中国（南昌）中医药科创城建设，力争完成中医药创新综合体建设。深入推进国家通用航空产业综合示范区建设，以洪都集团和瑶湖机场为依托，着力打造航空研发、航空制造、民航运输、通用航空、临空经济“五位一体”协同发展的产业体系。改造提升传统产业。大力实施“互联网+”、“标准化+”改造行动，着力提升食品、纺织等传统产业的产品、技术、工艺、装备现代化水平。做大做强产业集群。做大做强十大特色产业集群，培育发展新的产业集群，力争形成2个500亿级、2个300亿级、2个200亿级和4个100亿级的产业集群。扶优扶强龙头企业。着力培育一批核心竞争力强、带动作用明显、具有国际竞争力的大企业大集团，推动江铃集团成为千亿企业，江中集团、洪都航空、格特拉克成为百亿企业。

　　大力发展现代服务业。进一步加快中国（南昌）VR产业基地建设，抓好VR体验中心、VR产业展示馆等一批标志性项目建设，积极推动江西“VR+政务”试点示范应用。积极稳妥做好2018世界VR产业大会承办工作，推动VR产业迈上新台阶。以打造长江经济带重要消费中心和物流中心为目标，完成国家物流标准化试点工作，努力打造三位一体的江西省物流中心——向塘物流港。促进电子商务与传统制造业、服务业深度融合，推动电子商务相关交易额突破3000亿元；推进商圈转型升级，着力培育新洪城大市场、向塘物流基地等千亿商贸综合体，肉联集团30万吨冷链物流、绿滋肴等五百亿商贸综合体，苏宁广场、华润万象城、红星美凯龙爱琴海、宜家家居等百亿商贸综合体。着力打造绳金塔商旅文融合示范区、万寿宫历史文化街区、佑民寺历史文化街区、王府井新十字街（豫章古道）街区、长薪河商旅文街区等特色街区，大力发展楼宇经济。积极承办大型展会，做强做大会展经济。加快推进湾里国家全域旅游示范区、南矶湿地生态旅游区、大象湖城市休闲区、海昏汉文化旅游特色小镇、小蓝汽车特色小镇、大唐西市文化产业园、安义古村群民俗民宿聚落等项目建设，推进全域旅游发展。开展“天下英雄城·南昌”旅游品牌推介系列活动，放大南昌“三色”旅游资源优势，打造全国中小学生研学旅行重要目的地。

　　全面提升金融和人才服务实体经济的效率和水平。推动金融创新。加速全省金融商务区发展，打造全省金融发展核心增长极、中部区域性金融中心。推进多层次资本市场发展，大力推进企业上市、挂牌，有序发展股权融资，推动要素交易市场发展。发展壮大滕王阁城市产业发展基金，建立市县（区）全覆盖的国有出资融资性担保体系。开展“百家银行进千企”政银企对接活动，鼓励金融机构为普通投资者提供更多、更好的金融产品，引导更多金融资源配置到实体经济，促进金融和实体经济的良性循环。推动人才集聚。深入实施“洪城特聘专家”、“洪城计划”、“海鸥计划”、“211培训计划”等重点人才引进培训计划，推进洪城英才“五个一”工程、“洪燕领航”工程等人才选培项目，积极筹办中国南昌人才节、探索建设南昌国际人才城，不断充实“高精尖缺”人才队伍。以中国南昌人力资源服务产业园为平台，着力打造国家级技能人才实训示范基地。以“洪城工匠”为典型示范，带动各行各业树立工匠精神，打造百年经典企业。

　　2.聚焦农村与城市两大领域，不断拓展现代化经济发展的承载空间。

　　大力实施乡村振兴战略。按照“产业兴旺、生态宜居、乡风文明、治理有效、生活富裕”的总要求，制定全市贯彻落实乡村振兴战略行动计划。全面激发农业农村发展活力。重点实施“优质粮食工程”，深化粮食收储制度改革，高水平保障粮食安全。深化农村土地制度改革，积极探索“三权分置”的有效形式，稳妥推进土地经营权抵押贷款、土地经营权入股农业产业化经营、进城农民土地承包权依法自愿有偿退出等改革。深化农村集体经济产权制度改革，稳步推进农村集体经营性资产股份合作制改革，大力扶持发展村级集体经济。大力推进农业产业化发展，重点实施“农业产业九大工程”、打造“六大林下经济产业”。深入推进南昌绿谷“两区一廊”建设，沿城市外环线和紧邻互通出口处高标准规划建设20个左右“镇村园一体、一二三产互动、产加销对接”的田园综合体，加快推进溪霞现代农业园区、中国云厨（南昌）全域产业园、南昌菜园和安义千年古村群田园综合体等重点工程建设，深入推进“一乡一园”建设。积极培育新型职业农民、家庭农场、农民合作社、农业产业化龙头企业等新型农业经营服务主体，完善“龙头企业+农户”、“合作社+农户”的利益联结机制。积极培育壮大农村电商、休闲农业、智慧农业、大健康养老、“中央厨房”等新型业态，着力构建现代化农业产业体系、生产体系、经营体系。加快推动城乡融合发展。统筹城乡规划、基础设施、环境卫生一体化，完善公共资源优先向农业农村倾斜的政策体系，推动城乡水电路气网等基础设施互联，加快公共服务向农村覆盖，促进城乡公共服务均等化、公共资源均衡化。推动城市资本、技术、人才下乡，推进城乡要素双向融合互动。加快建设美丽文明乡村。坚持“规建管”并重、“点线面”并举、“生产空间、生活空间、生态空间”并优，持续推进“整洁美丽、和谐宜居”新农村建设，全面完成2000个左右新农村村点建设任务。推进21个特色小镇、30个左右“五位一体”综合示范村建设，探索小康示范村试点建设。引导健全村规民约，树立文明新风，完善自治、法治、德治相结合的乡村治理体系。

　　加快高品质现代化城市建设。坚持“一个尊重、五个统筹”，加快推进新城繁荣、老城复兴。坚持规划引领。加快推进城市总体规划修编，持续完善“多规合一”，大力推进南昌大都市区建设。 加快 “城市双修”“城市更新”等专项规划编制；加快推进传统村落和村镇建设全域规划。加快推进南昌市总体城市设计，统筹推进“一江两岸”、原省政府周边地区、“揽山入城”梅岭及周边地区、高铁东站周边地区等重点区域设计。完善主干交通网络。推动地铁2号线后通段区间贯通，加快推进3、4、5号线以及1号线北延至机场段的建设或前期规划工作。加快推进前湖大道、洪都大道、昌南大道、昌西大道等快速路改造工程。开工建设二七过江通道、艾溪湖隧道和滕王阁沿江隧道等快速通道工程。加快完成西二环高速公路、九龙湖过江通道、空港大道、高新大道快速路改造及南延工程、九洲高架快速路东延、天祥大道南延、广州路东延工程等项目前期工作。优化提升老城区。打好棚改旧改和城中村改造攻坚战，抓紧做好全市各征迁地块整体规划、项目开发和安置房建设等工作。分步实施老城区主干道立面提升改造工程，力争国庆前全面完成八一大道、北京路“一道一路”提升工程。繁荣发展新城区。进一步加快九龙湖新城、儒乐湖新城、瑶湖新城、望城新区、洪都新城、朝阳新城、昌南新城等区域功能设施配套建设。加快航空城市政设施等工程建设，深入推进朝阳、城南两个大型公交枢纽站建设，力争开工湾里、莲武路、凤凰洲、新洪城大市场公交枢纽站项目，继续深入推进“海绵城市”“地下综合管廊”建设和装配式建筑发展。

　　深入推动“美丽南昌·幸福家园”环境综合整治。对照“国考”标准，全力打赢全国文明城市“复牌”攻坚战。广泛深入开展“兴家风、淳民风、正社风”活动，进一步营造文明和谐、崇德向善的浓厚氛围。加快实施城市管理“六改”，推进城市家具微更新。加快实施城市生活垃圾分类试点工作，推进南昌固废处理循环经济产业园项目建设。建立健全农村生活垃圾治理长效机制，实现全市农村生活垃圾处理率达100%。继续深入开展停车场建设三年攻坚计划，力争全年新建公共停车场75个，同时实施“一点一策”，化解城市交通“堵点”问题。深化“厕所革命”行动，着力消灭农村旱厕，加快推进城乡公厕标准化、生态化、智能化。加快建设“智慧城市综合管理指挥中心平台”，提升城市综合管理数字化、智慧化、精细化水平。持续推进渣土运输、油烟排放、扬尘污染整治，深入开展秸秆禁烧工作。大力推进节地生态殡葬，开展丧葬陋习综合治理。加大养犬管理、禁止燃放烟花爆竹等法规的执法力度。

　　3.聚焦改革创新开放三大动力，不断激发现代化经济发展的活力来源。

　　持续推进全面深化改革。深化供给侧结构性改革，持续推进“三去一降一补”。深化“放管服”改革，持续深化商事制度改革，加快完善社会信用体系，加快推进相对集中行政许可权改革，让群众办事少跑腿或“一次不跑”。以完善市场经济体制为导向，深化国有企业改革，进一步探索混合所有制改革和员工持股等国企国资改革路径。实施优秀企业家成长计划，激发和保护企业家精神，鼓励、支持、引导民营企业做大做强。深化财税体制、价格体制、土地管理制度等各领域改革。

　　加快建设创新型城市。推进实施“一产一院”“一产一校”，组建产业创新联盟，支持大院名校与我市共建或者企业自主建设新型研发机构，打造更多国家级、省级科技创新平台。统筹推进南昌（国家）大学科技城建设，加快建成科技广场，搭建集信息网络服务、“一站式”科技创新创业服务为一体的科技创新公共服务平台。积极培育国家级高新技术企业，力争新增国家级高新技术企业突破680家。大力推进“知识产权示范城市”“首批全国重点产业知识产权运营服务试点城市”“国家第二批促进科技和金融结合试点城市”建设，高标准建成“南昌知识产权保护中心”。深入打造“洪城众创”空间，建立较为完整的创新创业孵化体系，培育一批国家级、省级“星创天地”；开展“洪城之星”“创业故事汇”等创新创业活动，掀起“双创”新一轮热潮。

　　加快推进双向开放。强化精准招商。瞄准各产业环节的领袖企业、关键企业，开展产业链招商，引进一批支撑性的补链强链扩链型项目；以2018年江西省战略性新兴产业推介会、赣港会、赣台会、绿发会等为契机，引进一批高精尖项目；全力承接北京非首都功能疏解和产业转移，积极对接央企入赣和赣商回归投资。扩大对外开放。积极对接“一带一路”，引导优势企业产能出海、境外投资、跨国并购，加快培育一批具有国际竞争力的本土跨国公司。推进国际友城交流，深化各领域合作，积极争取承办专业化、高端化的国际会议和国际论坛。深化区域合作。按照共抓大保护、不搞大开发的要求，积极推动长江经济带发展。深度融入“长珠闽”经济板块，着力加强与闽浙赣皖福州经济协作区、泛珠三角、长江中游城市群的联动发展；继续大力支持和配合赣江新区建设，推进昌九、昌抚一体化。完善开放平台。加强南昌综合保税区与机场、码头、铁路等口岸平台互动，加快推进南昌铁路口岸和指定口岸建设；积极推进中欧（南昌—鹿特丹）、赣欧（亚）南昌—河内国际铁路货运班列和海铁联运健康发展；推广国际贸易单一窗口，提高贸易自由化便利化水平，优化通关环境。

　　（二）牢牢把握三大攻坚战的重点任务，坚持底线思维，为决胜全面建成小康社会提供有力保障

　　打好防范化解重大风险攻坚战。严格规范地方政府举债行为，完善政府债务考核，强化融资平台公司监管，稳妥处置隐性债务存量，有效遏制隐性债务增量，切实防范和化解政府债务风险。强化金融风险监测预警，提高风险防范和应急处置能力。大力整治各类金融乱象，严厉打击乱办金融、非法集资等违法违规金融活动，深入开展互联网金融风险专项整治，有效防范处置银行不良贷款，维护金融市场稳定。完善金融制度建设，强化企业资产负债率和资本金约束，加强社会信用体系建设。完善地方金融执法监督工作体系，加强对影子银行、互联网金融等薄弱环节的监管，坚决守住不发生区域性金融风险的底线。坚持房子是用来住的、不是用来炒的定位，落实地方主体责任，完善调控机制，合理引导市场预期和购房行为，防止房价出现大的起落。严格限制信贷资金用于投资投机性购房，坚决打击房地产领域非法集资活动。完善土地、税收、金融等措施，建立健全房地产发展长效机制，大力发展住房租赁市场，加快建立多主体供应、多渠道保障、租购并举的住房制度，促进房地产市场平稳健康发展。

　　打好精准脱贫攻坚战。加大扶贫政策落实力度，巩固“两不愁、三保障”成果。大力实施产业扶贫全覆盖、搬迁扶贫再精准、健康扶贫再提升、教育扶贫再对接、贫困村村庄整治再推进工程。大力实施“六个一批”工程，培育发展贫困村特色种养、休闲旅游、农村电商、光伏扶贫四大产业。深入开展“机关单位挂点帮扶”“百企扶百村”行动，广泛动员社会力量参与精准脱贫，倡导乡贤“致富思源”做公益慈善。注重扶贫与扶志、扶勤、扶德、扶智并举，充分激发脱贫发展的内生动力。严格考核评估，强化督查问责，做到真脱贫、脱真贫，确保7921人减贫、22个贫困村退出。

　　打好污染防治攻坚战。坚持全民共治、源头治理，持续开展“蓝天、清流、净土”三大行动，抓好中央环保督察问题整改和“回头看”。深入贯彻落实“气十条”，开展城市“四尘”“三烟”“三气”专项治理，坚决打赢蓝天保卫战。深入贯彻落实“水十条”，全面建成工业园区污水设施，大力整治城市黑臭水体，全面消灭6个劣Ⅴ类水质断面，保护河湖及支流水质，保障群众用水安全。深入贯彻落实“土十条”，完善土壤污染风险管控机制，加强固体废弃物和垃圾分类化、减量化、资源化处理，扎实开展土壤污染治理和修复。大力开展山水林田湖草综合治理，加快推进造林绿化和退耕还林、森林质量提升、湿地保护与修复、水土流失综合整治等绿色生态工程建设，持续提高生态系统质量和稳定性，增加并优化生态产品供给，永葆“南昌蓝、梅岭绿、赣江清”。在此基础之上，坚持生态优先、绿色发展，加快营造人与自然和谐共生的生态环境。完善生态文明制度。深化生态文明体制改革，完善“一委四组三机制”，加快推进环保监测监察执法垂直管理制度改革，支持检察机关提起公益诉讼工作，进一步理顺生态环境保护机制体制。严守生态保护红线、永久基本农田、城镇开发边界三条控制线，加快推行全域“多规合一”，实现国土空间集约、高效、可持续利用。坚持“谁受益、谁补偿”，加快推进森林、湿地、水流、耕地四个重点领域生态保护全覆盖。建立健全自然资源开发保护制度体系，全面开展生态文明综合考核，编制自然资源资产负债表，全面推行领导干部自然资源资产离任审计，落实生态环境损害责任追究制度，着力提高政府治理环境、保护生态的总体水平。提升绿色发展水平。坚持“生态+”理念，积极推行低碳化、循环化、集约化发展模式。加快推进工业多层标准厂房、下沉式厂房建设，新开工面积300万平方米以上。大力推进生态环保产业发展，加快培育节能工业、绿色建筑、生态农业。开展生态文明创建活动，倡导绿色低碳的生活方式和消费方式，让绿色成为南昌发展的底色。

　　（三）牢牢把握人民对美好生活的向往，坚持以人民为中心的发展思想，加快创建共建共治共享的现代化社会治理体系

　　优先发展教育事业。深化教育体制机制改革，主动适应新高考改革，推进中考制度改革。加强师风师德建设，继续实施校长教师交流轮岗制度，深入实施教育集团发展带动战略，优化配置中小学校教师资源，完善教师“县（区）管校用”管理机制。完善中小学网点布点规划，实施新扩建项目51个。建立“幼有所育”体系，着力解决婴幼儿照护和儿童早期教育服务问题。实施第三期学前教育行动计划，支持建设普惠性民办幼儿园，增加公办普惠学前教育资源供给。完成“全面改薄”，重点推进新建区、进贤县义务教育均衡发展，实现全市义务教育均衡发展全面达标。继续巩固“零择校”成果，精准施策解决中小学生课外负担重、“大班额”等突出问题。实施优质高中建设工程，启动豫章中学红角洲校区建设和十五中、十六中、三十中改扩建项目。积极办好特殊教育和网络教育。筹建南昌健康职业技术学院。加快高等教育内涵式发展，推进豫章师范学院实验实训大楼项目建设。深化产教融合与校企对接，健全特色职业教育和培训体系。

　　实施就业优先战略。大力实施就业促进、创业引领、基层成长计划，推进大学生留昌返昌就业创业。推广“信息化+就业服务”新模式，拓宽下岗职工、农村转移劳动力与城镇就业困难人员的就业渠道，确保城镇就业率达95%，零就业家庭就业安置率达100%。构建“六位一体”青创服务体系。完善创业担保贷款机制，以创业带动就业。深化收入分配制度改革，多渠道增加城乡居民劳动收入和财产性收入，引导更多人特别是青年人靠劳动致富、靠知识致富、靠创新致富。

　　加快健康南昌建设。深化医药卫生体制改革特别是城市公立医院综合改革，积极稳妥推进分级诊疗和家庭医生签约服务，有效缓解“看病难、看病贵”问题；加快城市紧密型医联体、县乡村医疗卫生服务共同体、专科联盟、远程医疗协作网建设，探索医疗集团化改革。抓好卫生防疫，加强传染病疫情等突发事件应急处置工作，推进慢性病综合防控示范区创建。加快推进市洪都中医院新院二期等6个项目建设，配合推进省妇幼保健医院等5所省属新院竣工。坚持中西医并重，推进中西医“三个中心”建设。坚持“三不变、四到位”，完善计划生育长效工作机制。加快全民健身设施建设，实施足球改革发展行动计划，办好2018南昌国际马拉松，继续保持竞技体育在全省领先优势。加快国家医养结合试点城市建设，推进全国居家和社区养老服务改革试点工作，推动医疗服务与养老、旅游、互联网、体育、食品深度融合发展，不断满足群众多元化多样化的健康需求。

　　完善社会保障体系。全面实施全民参保计划。加大社保缴费清欠稽核力度，实施社保“统受分办”经办模式改革，不断提高社会保险基金安全和使用效益。推进医疗保险纳入省级统筹盘子，探索建立长期护理保险制度，全面实施城乡居民大病保险制度。进一步完善失业、工伤、生育保险制度。严格执行“救急难”运行机制，着力构建“弱有所扶”体系。健全农村留守儿童和妇女、老年人关爱服务体系。落实残疾人保障扶持政策，加强残疾人康复服务。加快市老年福利院、青少年宫开放运营。出台“零点行动”方案，加快推进殡葬综合改革。大力支持国防和军队改革建设，制定军民融合发展工作方案，扎实做好优抚安置、拥军优属、国防动员、人民防空等工作，力争实现全国双拥模范城“九连冠”。

　　加快建设文化强市。深化文化体制改革，大力实施中华优秀传统文化传承发展工程，出台加快文化强市建设的工作意见，继续推进国家文化消费试点城市建设，推动文化事业和文化产业创新发展。加快推进文化惠民工程，启动公共图书馆信息化建设“香樟树”工程，完成南昌“公共文化云”二期工程，建成500个行政村（社区）基层综合性文化服务中心。启动市档案馆建设。加强文化遗产保护传承和文物保护利用，大力弘扬南昌采茶戏，启动海昏侯刘贺墓园保护展示、环境整治工程，建设全国八一少年军校基地。推进文化与科技、信息等深度融合，布局实施一批出版创意、数字出版、网络视听等重大产业项目。完成小平小道陈云旧居陈列馆提升改造工程二期项目，隆重举办我市纪念改革开放40周年系列活动。

　　加强和创新社会治理。全面推进法治南昌、平安南昌建设。完善社会治理制度，加快诚信体系建设。加快构建以人民为中心的公共法律服务体系。提高社会治安防控智能化水平，深化“数据强警”建设。强化基层基础、分类管控、应急准备、专群结合，坚决捍卫国家安全。健全矛盾风险预警和化解机制，加大对房屋征迁、环境投诉、传销诈骗、权益保障等突出问题化解力度。落实落细安全生产责任制，完善消防、建筑装修、道路交通、危爆物品等重点领域的安全监管机制，坚决遏制重特大安全生产事故发生，加强应急体系建设，提升防灾减灾救灾能力。深入系统推进国家食品安全示范城市创建工作，强化食品安全属地责任考核，探索建立重点食品药品追溯体系，确保“舌尖上的安全”。完成第十届村（居）委会换届选举，提高城乡社区协商民主水平。激发社会组织活力，支持工、青、妇、红十字会等群众组织开展工作。全面贯彻党的宗教政策，积极推动民族团结进步。

　　各位代表！迈步新时代，完成新目标，回应新期待，必须进一步加强政府自身建设。我们将坚持把党的政治建设摆在首位，牢固树立“四个意识”，坚决维护习近平总书记在党中央、在全党的核心地位，坚决维护以习近平同志为核心的党中央权威和集中统一领导，坚决贯彻落实党中央、国务院、省委、省政府和市委的各项决策部署，确保政令畅通、令行禁止，切实增强政府系统的思想自觉、政治自觉、行动自觉。提升学习力、操作力。既要政治过硬，也要本领高强。精准领悟并践行习近平新时代中国特色社会主义经济思想，培养专业能力，弘扬专业精神，深入研究加强地方政府债务管理、发展新经济培育新动能等重大课题；坚持问题导向，敢于攻堡垒破难题，善于补短板强弱项，勇于创亮点争进位。提升执行力、公信力。认真执行人大决定，自觉接受人大及其常委会、政协、监察、司法、社会、舆论等各方面的监督。完善政务清单管理制度，持续推进政务公开和政府信息公开。提高12345政府服务热线平台运行效率，认真对待并切实解决群众反映强烈的问题。提升约束力、战斗力。强化党风廉政建设“两个责任”，严格遵守中央八项规定实施细则，落实“一岗双责”，加大整治“四风”问题力度，聚焦侵害群众切身利益的“微腐败”，做到有腐必惩、有贪必肃。完善政府部门和干部考核评价机制，强化责任落实、督查检查和绩效考核。大力弘扬苦干实干作风，为南昌发展勇担当，为全市人民谋福祉。

　　各位代表！新时代赋予新使命，新征程呼唤新作为。让我们紧密团结在以习近平同志为核心的党中央周围，在省委、省政府和市委的坚强领导下，不忘初心，牢记使命，担当拼搏，开拓进取，为打造富裕美丽幸福现代化江西“南昌样板”而努力奋斗！
